# Demographical and Clinical Characteristics of Behcet’s Disease in Southeastern Turkey

**DOI:** 10.14740/jocmr1952w

**Published:** 2014-09-09

**Authors:** Bilal Sula, Ibrahim Batmaz, Derya Ucmak, Ilyas Yolbas, Sedat Akdeniz

**Affiliations:** a Department of Dermatology, Dicle University Medical School, Diyarbakir, Turkey; b Department of Physical Medicine and Rehabilitation, Dicle University Medical School, Diyarbakir, Turkey; c Department of Pediatrics, Dicle University Medical School, Diyarbakir, Turkey

**Keywords:** Behcet’s disease, Epidemiology, Southeastern Turkey

## Abstract

**Background:**

We aimed to determine the demographic and clinical features of patients with Behcet’s disease (BD) in Southeastern Turkey.

**Methods:**

In this study, files of 132 patients with BD (76 females and 56 males) who were diagnosed with BD according to the International Study Group criteria at the Department of Dermatology of Dicle University Faculty of Medicine from 2005 to 2009 were evaluated retrospectively. Demographical and clinical characteristics of the cases were recorded.

**Results:**

Mean age of the cases was 32.40 ± 9.4 years (range 15 - 59 years) and male/female ratio was 0.73. The mean age at diagnosis was 28.71 ± 9.1 years. Six cases were diagnosed as juvenile BD (4.45%). Oral aphthous lesions (100%) and genital ulcers (94%) were found to be the most common findings of the disease, followed by pathergy positivity (75%), papulopustular lesions (74.2%), erythema nodosum (43.2%), thrombophlebitis (6.8%) and extragenital ulcers (6.1%). Systemic involvement was noted as joint involvement in 79.5%, ocular involvement in 28.8%, vascular involvement in 9.8%, pulmonary involvement in 2.3%, neurologic involvement in 2.3% and genitourinary system involvement in 0.8%. There was no significant difference between mucocutaneous findings and systemic involvement ratios of male and female cases.

**Conclusion:**

Demographic and clinical features of BD may vary according to geographical region, gender and ethnicity. We hope that this study will contribute to the epidemiologic data of BD which may exhibit different clinical and demographic features in different parts of the world.

## Introduction

Behcet’s disease (BD) is a chronic multisystemic disorder characterized by oral ulcers, genital ulcers, cutaneous lesions, ocular, articular, neurological and vascular involvement. BD most often affects patients in their second or third decade, but can occur at any age. There seems to be a little male predominance but this finding varies from country to country. Although this predominance is seen almost all over the world, it is more common in Turkey, Iran, Japan, Korea and China [1-4]. In previous studies from Turkey, the prevalance of BD was reported as 8-37/10,000 [5]. We aimed to determine regional specific findings of BD in Southeastern Anatolia.

## Methods

Files of 132 patients who were diagnosed by the criteria of the International Study Group [6] of BD at the Department of Dermatology of Dicle University Faculty of Medicine from 2005 to 2009 were evaluated retrospectively. Patients’ age, gender, age at diagnosis, duration of disease, age at onset of oral aphthous lesions and genital ulcers, localization of the lesions, skin manifestations (such as erythema nodosum, papulopustular lesions, superficial thrombophlebitis, extragenital ulcers and pathergy test), ophthalmologic, joint, neurological, vascular and other systemic involvement were recorded. In addition, findings of clinical evaluation of symptoms and opthalmological screening every 6 months were noted. SPSS 16 was used to record and evaluate the data.

## Results

Our study included 132 cases whose ages ranged from 15 to 59 years. The mean age of cases was 32.4 ± 9.4 years. The mean ages of males and females were 34.57 ± 8.68 years and 30.8 ± 9.51 years, respectively (P < 0.05). Seventy-six (57.6%) cases were female and 56 (42.4%) cases were male. Male/female ratio was 0.73. The most common age range was between 26 and 35 years (39.4%). Mean age is lower in female patients in BD. A comparison of the sexes for age revealed a statistically meaningful difference (P < 0.05).

Age at onset of symptoms was 24.2 ± 9.1 years in females and 27.2 ± 8.4 years in males (P < 0.05). Age at onset of symptoms ranged from 8 to 54 years. Duration of disease in patients ranged from 1 to 16 years. The average duration of illness was 4.05 ± 2.9 years. The mean age at diagnosis was 28.71 ± 9.0 years. The ages at diagnosis of males and females were 30.8 ± 8.55 years and 27.17 ± 9.22 years, respectively (P < 0.05). Female and male patients were compared for the initial age of symptoms of the disease, with statistically meaningful differences (P < 0.05).

Comparison of the ages of the patients by sex and the initial age of the symptoms, duration of the disease and diagnosis ages for BD is summarized in Table 1. The distributions of clinical findings in male and female patients are shown in Table 2.

**Table 1:** Comparison of the Ages of the Patients by Sex and the Initial Age of the Symptoms, Duration of the Disease and Diagnosis Ages for Behcet’s Disease

	Male	Female	P
Age (years)	34.57 ± 8.68	30.8 ± 9.51	0.02
Initial age of symptoms	27.25 ± 8.48	24.22 ± 9.17	0.05
Duration of the disease	3.77 ± 2.92	3.62 ± 2.96	0.77
Diagnosis age	30.8 ± 8.55	27.17 ± 9.22	0.02

**Table 2:** The Distribution of Clinical Findings of Patients With Behcet’s Disease According to Gender

Findings	Male (n/%)	Female (n/%)	Total (n/%)
Oral aphthae	56 (100%)	76 (100%)	132 (100%)
Genital ulcer	52 (93%)	72 (95%)	124 (94%)
Erythema nodosum	19 (34%)	38 (50%)	57 (43.2%)
Papulopustular lesions	43 (76.7%)	55 (72.3%)	98 (74.2%)
Thrombophlebitis	4 (7.1%)	5 (6.5%)	9 (6.8%)
Pathergy test	42 (75%)	57 (75%)	99 (75%)
Eyes involvement	19 (33.9%)	19 (25%)	38 (28.8%)
Joint involvement			
Arthralgia	40 (71%)	60 (79%)	100 (76%)
Arthritis	10 (18%)	20 (26%)	30 (23%)
Sacroilitis	3 (5%)	6 (8%)	9 (7%)
Vascular involvement	6 (10.7%)	7 (9.2%)	13 (9.8%)
Neurological involvement	2 (3.5%)	1 (1.3%)	3 (2.3%)
Pulmonary involvement	2 (3.5%)	1 (1.3%)	3 (2.3%)
Genitourinary involvement	1 (1.8%)	0	1 (0.8%)

Patients with initial symptoms at age 16 years or younger were considered as having juvenile BD. In our study, the prevalence of juvenile BD was found to be 4.54% among patients with BD. Clinical features of juvenile cases are shown in Table 3.

**Table 3:** Clinical and Demographical Features of Juvenile Behcet’s Disease

Patient	Age of diagnosis	Gender	OA	GU	EN	PPL	Pathergy	Thrombophlebitis	Eye involvement
1	11	M	+	+	+	+	+	+	-
2	14	F	+	-	-	-	+	-	+
3	14	F	+	+	+	+	+	-	-
4	14	F	+	-	-	+	+	-	+
5	15	M	+	+	-	+	+	-	-
6	15	F	+	+	-	-	+	-	-

OA: oral ulcer; GU: genital ulcer; EN: erythema nodosum; PPL: papulopustular lesions.

Oral aphthae was observed in all of the 132 patients (100%). Oral aphthae was found in the form of minor ulcer in 73 (55.3%) patients, major ulcer in seven (5.3%) patients, and minor and major ulcer in 52 (39.4%) patients. The number of lesions was less than 5 in 93 (70.5%) patients, and between 5 and 10 (27.3%) in 36 patients. The frequency of attacks was found between 3 and 5 in 100 (75.8%) patients. A comparison of male and female patients for the number of oral aphthae showed less than five lesions in 43.4% of males and 79% of females. This difference was found to be statistically significant (P < 0.05). No statistically significant differences were found among sexes for the size of lesions, improvement time and frequency of attacks.

Genital ulcer was determined in 124 patients (94%). Genital ulcers were detected in 93% (52/56) of male patients and 95% (72/76) of female patients. The initial symptom of BD was genital ulcer in three of the female patients. The most frequent locations of genital ulcers were 85% in the scrotum in the male and labiums and vulva in the female. There was genital scarring due to previous lesions in 55.3% of the patients. The prevalence of extragenital ulcers was found 6.1%. A comparison of male and female patients for the number and size of genital ulcer lesions, and the frequency of attacks found no statistically meaningful difference (P > 0.05) between them.

Forty-three (32.6%) patients had a single genital ulcer lesion, and 81 (61.3%) had two or more genital ulcer lesions. Fifty-five (41.7%) patients experienced one attack, and 69 (52.2%) patients experienced two and more attacks annually. Seventy-three (55.3%) patients had scar tissues of former lesions. Cicatricial lesions of former lesions were seen more in male patients than in female patients and this difference was found to be statistically meaningful.

Erythema nodosum was found in 57 (43.2%) patients. Erythema nodosum was more common in female patients, with no statistically meaningful difference (P > 0.05).

Papulopustular lesions were found in 43 (76.7%) male and 55 (72.3%) female patients. A positive pathergy test was found in 42 (75%) male patients, and 57 (75%) female patients. No statistically meaningful differences were found between the sexes for papulopustular lesions and positive pathergy.

Less common skin findings such as furunculosis in two male patients, Sweet’s syndrome-like lesions in one male and in one female, leg ulcers and stasis dermatitis in one male patient, and recurrent erythema multiforme-like lesions in one female patient were noted.

Ocular involvement was noted in 38 (28.8%) patients (panuveitis in 25 (18.9%) patients, anterior uveitis in six (4.5%) patients, posterior uveitis and iridocyclitis in two (1.5%) patients, and intermediate uveitis, retinal vasculitis, retinal detachment and keratitis in one (0.8%) patient). Ocular symptoms such as decreased vision, burning, pain, itching and redness were found in 24.2% of patients.

Of 105 patients (79.54%) with joint complaints, monoarthralgia was seen in 21 (15.9%) patients, oligoarthralgia in 39 (29.5%), polyarthralgia in 40 (30.3%), monoarthritis in 24 (18.2%), oligoarthritis in five (3.8%), and poliathrtitis in one (0.8%).

Deep vein thrombosis was seen in five (3.8%) patients (one female and four males). There was superior vena cava thrombosis, superior vena cava syndrome and pulmonary thromboembolism in a female. There was osteonecrosis of the femoral head and abdominal aortic aneurysm in a female. A 22-year-old male with pulmonary involvement died due to massive pulmonary hemorrhage.

Sixty-two (47.0%) patients had gastrointestinal tract complaints such as abdominal pain, indigestion, heartburn, diarrhea, nausea and vomiting, melena and/or hematochezia and weight loss.

When family history was evaluated, 15 (11.4%) patients had BD, 24 (18.2%) patients had recurrent aphthous stomatitis (RAS) and four (3.0%) patients had both BD and RAS in their family history.

## Discussion

BD is most common in young adults between the second and fourth decades. Although it can begin at any age, it is rare in children and adults over 50 years of age. The average age of onset of BD has been reported as 25.6 years old in Turkey [7-9].

The age at onset of first symptoms and age of diagnosis of BD may vary. Uslu et al found the average age of onset of symptoms as 27.1 years, and Karincaoglu et al found the average age of onset of symptoms between 16 and 25 years in their study [10, 11]. In our study, most of the patients’ ages were in the 26 - 35 age group and the average age of onset of symptoms was found to be 25.5 years. Uslu et al reported the average age of diagnosis as 32.4 years [10]. In our study, the average age of diagnosis was 28.7 years.

Although most studies report that BD is more common in male, gender rate may vary from study to study [5, 7]. Tursen et al found the male/female ratio as 1.03, Karincaoglu et al found male/female ratio as 1.6, and Uslu et al found male/female ratio as 1.48 [10-12]. In our study male/female ratio was found to be 0.73.

Skin lesions are common in BD. Oral ulcers is the most common and unchanging symptom of the BD. It is seen as the first symptom in 70% of patients, and prevalence of oral ulcers in patients with BD was reported in Turkey to be 100%, in Iran 96.8%, in Japan 98.2%, in Korea 97.5%, in Morocco 100% and in UK 100% [10, 13]. Also the prevalence of genital ulcers in patients with BD was reported in Turkey to be 85.3-97% [10-12, 14], 73% in Japan and 65-91% in Europe [9, 13, 15]. In our study, the prevalence of oral ulcers was found to be 100% and the prevalence of genital ulcers was 94%. These results were similar to previous studies conducted in Turkey. In addition, oral ulcers were detected in 97.8% of patients as the initial symptom and genital ulcers were detected only in 2.2% of patients as the initial symptom. This result may be due to the fact that patients who have genital ulcers generally do not go to the hospital because of shame or fear or intimacy or disregard. For this reason patients are usually diagnosed with oral ulcers as the first symptom of BD. The most frequent locations of genital ulcers were 85% in the scrotum in the male and labiums and vulva in the female. There were lesions of old scar tissue in 55.3% of the patients. The prevalence of extragenital ulcers has been reported as 2% in Korea, 1% in China and 0-13% in Europe [15]. In our study it was found to be 6.1%.

In previous studies, prevalence of papulopustular lesions was reported in Turkey as 54-72.3%, 40% in China, and 41-49% in Europe [15]. In our study, the prevalence of papulopustular lesions was found as 74.2%.

In different studies, prevalence of erythema nodosum-like lesions was reported as 15-78% (in Turkey 47%, in China 64%, and in Europe 25-78%) [15]. In our study, the prevalence of erythema nodosum was found to be 43.2%. The most common localization was the distal lower extremities, and the upper extremities were less frequently affected.

Prevalence of positive pathergy test of patients with BD was found to be 50-80% in Turkey, 44% in Japan, and 12-52% in Europe [15, 16]. In our study, the prevalence of positive pathergy test was 75%. This reason may be associated with some factors such as the test being performed at active periods of the disease, when most patients were not receiving any systemic treatment, when cleansers were not used prior to application and use of 20 G needles.

Eye involvement, which is one of the most important organ involvement, is 40-70% in patients with BD [17]. Eye involvement is more frequent and more severe in males and young adults; however, it is very rare and less severe in females and the elderly [3, 12, 16, 18]. Prevalence of eye involvement was reported in Turkey to be 21.5-39%, 69% in Japan, 40% in China, 59% in Iran and 35-69% in Europe [15]. In our study, the prevalence of eye involvement was found as 28.8%. Prevalence of joint involvement was reported to be 50-90% [19, 20]. In our study the prevalence of joint involvement was found to be 79.5%.

Neurological involvement is usually seen in the first 5 years, it has a high risk of morbidity and mortality, and it occurs more commonly in males with BD [3, 18]. Prevalence of neurological involvement is 5-10% in the world, 2.3-7.6% in Turkey, 11% in Japan, 2% in China and 11-48% in Europe [7, 16]. In our study, the prevalence of neurological involvement was found to be 2.3%.

Prevalence of vascular involvement was reported by Alpsoy et al as 4.4%, Tursen et al as 7%, Uslu et al as 9.1%, Karincaoglu et al as 16.1% [10-12, 14]. In our study, the prevalence of vascular involvement was found to be 9.8%.

The actual prevalence of pulmonary involvement is not known. Prevalence of vascular involvement was reported in Turkey to be 1-1.1%, in Europe 0-17%, and in Taiwan 3% [15]. In our study, the prevalence of pulmonary involvement was found to be 2.3%.

Prevalence of gastrointestinal involvement varies in different societies such as 1.4-5% in Turkey, 14% in France, 14% in Britain and 50-60% in Japan [21]. In our study, however, no gastrointestinal involvement was detected, although 47% of the patients had gastrointestinal complaints such as abdominal pain, dyspepsia, heartburn, diarrhea, nausea and vomiting, melena and/or hematochezia and weight loss.

Involvement of the genitourinary system is seen in about 5% of patients with BD [13, 18, 19]. Karincaoglu et al reported the prevalence of genitourinary system involvement as 9.3% [11]. In our study, genitourinary system involvement was seen in only one patient (0.8%) who had orchitis.

Juvenile BD is rare, and it is used to define patients under 16 years who satisfy the diagnostic criteria of the disease. Patients with juvenile BD comprise 3-7% of all patients with the disease. Among patients with BD, the prevalence of juvenile BD was reported in Turkey to be 2-10.5%, and 1.6% in Japan [22-24]. In our study, the prevalence of juvenile BD was found to be 4.54% among patients with BD.

### Conclusion

Our study revealed no significant differences between male and female patients for mucocutaneous (genital ulcer, papulopustular lesions, erythema nodosum, extragenital ulcer and superficial thrombophlebitis) and systemic involvements. The study found that our patients had a higher incidence of mucocutaneous findings, with no meaningful difference between the sexes. The study found a higher incidence of joint complaints and sacroiliitis among female patients, with more common eye involvement in male patients. However, these differences were not considered significant. The study found a significantly high level of headache in female patients. This study found that our patients with BD had parallel demographic and clinical findings with previous studies, with no meaningful difference.

In conclusion, the demographic and clinical features of BD may vary according to different geographical regions, gender and ethnicity. Mucocutaneous findings were more common in our patients, which is relatively similar to other studies conducted in Turkey. We hope that this study will contribute to the epidemiology of BD that may vary in clinical and demographic features in different regions of the world.
